# Unsecured debt in early adulthood and premature mortality in adults in the USA: a longitudinal analysis of prospective national cohort data

**DOI:** 10.1016/S2468-2667(25)00226-9

**Published:** 2025-11

**Authors:** Samuel L Swift, Zihan Chen, Calvin Colvin, Katrina Kezios, Sebastian Calonico, Adina Zeki Al Hazzouri

**Affiliations:** College of Population Health, University of New Mexico, Health Sciences Center, Albuquerque, NM, USA (S L Swift PhD); Department of Epidemiology, Mailman School of Public Health, Columbia University, New York, NY, USA (Z Chen MA, C Colvin MSPH, A Zeki Al Hazzouri PhD); Department of Epidemiology, Boston University School of Public Health, Boston, MA, USA (K Kezios PhD); Graduate School of Management, University of California in Davis, Davis, CA, USA (S Calonico PhD)

## Abstract

**Background:**

Premature mortality rates are higher in the USA than other peer nations. Few studies have assessed the association between cumulative unsecured debt and subsequent premature mortality. The aim of this study was to investigate the association between cumulatively accrued unsecured debt over 20 years of early adulthood and subsequent premature mortality in midlife (age 41–62 years).

**Methods:**

For this longitudinal analysis, we used data from 6954 participants included in the US National Longitudinal Survey of Youth 1979. Participants were followed up from 1985 to 2004 to assess debt trajectory, and from 2004 to 2018 to assess premature mortality. A group-based trajectory model was used to classify four groups of unsecured debt trajectories: no debt, constant low debt, constant medium debt, and increasing debt. Multivariable adjusted Cox proportional hazards models were used to assess associations between debt trajectory and mortality.

**Findings:**

Of the 6954 participants included in our analysis, 5670 (81·5%) individuals had constant low debt, 712 (10·2 %) had constant medium debt, 148 (2·1%) had increasing debt, and 424 (6·1%) had no debt. In adjusted models, the risk of mortality was 89% higher in the increasing debt group than the constant low debt group (hazard ratio 1·89 [95% CI 1·14–3·12]). In unadjusted models, individuals with no debt had a numerically higher risk of premature mortality compared with those with constant low debt; however, this difference was not statistically significant.

**Interpretation:**

Cumulative increasing unsecured debt in early adulthood was associated with increased risk of premature mortality in midlife. Interventions and policies targeting unsecured debt might reduce premature mortality.

**Funding:**

National Institute of Health National Institute on Aging.

## Introduction

Since the 1990s, life expectancy in the USA has remained lower than other comparable high-income countries.^1^ The lower life expectancy in the country is partly driven by higher premature mortality in midlife (age 25–64 years) than other countries,^2^ and this disparity has been attributed to socioeconomic causes.^3^ Previous research has assessed the association between socioeconomic drivers such as income and education and premature mortality;^4^ however, the association between debt and premature mortality remains underexplored.

Within the USA, the 1980s and 1990s were marked by financial deregulation and expanded access to credit.^5^ Household debt (ie, secured debt and unsecured debt) has increased substantially in the USA since the 1980s.^6^ Unsecured debt, which can be defined as debt that is not tied to an asset such as a house or car, might include credit cards, payday loans, student loans, and money owed to businesses.^7^ In contrast, secured debt is linked to assets such as homes or cars, and therefore might reflect a person’s wealth. Unsecured debt carries higher interest rates than secured debt,^7^ and generally does not contribute to wealth accumulation. This category of debt might be more stressful and burdensome than other types of debt, and therefore might be particularly important to study as a social determinant of premature mortality.

Previous research has established strong associations between types of unsecured debt and a wide range of health outcomes, demonstrating that individuals with high levels of unsecured debt (high interest loans, credit card debt, medical debt) are more likely to experience physical^8–12^ and mental health symptoms.^9,12–15^ A meta-analysis of 65 studies on the association between unsecured debt and health outcomes found that unsecured debt has a particularly strong association with mental health outcomes, substance abuse, and suicide outcomes, including suicide completion.^12^ However, few studies have used data from nationally representative longitudinal cohort studies,^12^ and even fewer studies have examined the association between debt and premature mortality. Specific to mortality, studies using aggregate data found that country-level unsecured debt in Europe was associated with more years of potential life lost^16^ and county-level medical debt in the USA was associated with increased premature mortality.^17^ Additionally, an economic working paper^18^ found that delinquent debt (ie, debts more than 90 days overdue) in the USA was associated with higher overall mortality. Most previous cohort studies have measured debt at a single timepoint,^12^ or for a short period,^13^ which does not capture long-term trends of unsecured debt accumulation and its potential effect on health. Two previous studies in the National Longitudinal Survey of Youth (NLSY) found unstructured debt was associated with joint pain^8^ and mental health outcomes^19^ in midlife.

The present analysis builds on previous research to assess the association between the trajectory of unsecured debt and premature mortality. To our knowledge, to date, no studies have investigated the association between unsecured debt trajectory and premature mortality using data from a large and nationally representative longitudinal US cohort. In this study, we aimed to investigate the association between cumulatively accrued unsecured debt over 20 years of early adulthood and subsequent premature mortality in midlife (defined as age 41–62 years in the present analysis) among adults in the USA. In this study, we aimed to elucidate the association between unsecured debt and premature mortality.

## Methods

### Study design and participants

For this longitudinal analysis, we used data from the NLSY 1979 (NLSY79), a nationally representative survey sponsored and directed by the US Bureau of Labor Statistics and conducted by the Center for Human Resource Research at Ohio State University. The NLSY79 was launched in 1979 and enrolled 12 686 participants aged 14–21 years, with face-to-face interviews conducted annually from 1979 to 1994, and biennially thereafter until 2018. Further detailed methods of this cohort have been published elsewhere.^20^ Individuals eligible for inclusion in our analysis were part of the initial survey in 1985; were not part of a discontinued NLSY79 subsample^20^ (n=9986); had data on unsecured debt in 1985 and at least two additional waves during our exposure period (n=9333); remained in the NLSY79 survey by 2004 (n=7452); and had complete data on covariates (appendix p 1). These exclusions resulted in a final analytical sample of 6954 participants.

### Exposures

Unsecured debt was assessed annually from 1985 to 1990, and from 1992 to 1994, biennially from 1996 to 2000, and in 2004 (13 waves in total). Using self-reported survey questions, unsecured debt was initially measured from 1985 to 2000 by asking participants, “Do you or your partner owe over US$500 to any stores, hospitals, banks, or anyone else?” In 2004, the survey began distinguishing unsecured debt into four categories: credit card debt, debt owed to other individuals, business debt, and medical debt. However, due to data availability limitations, we were unable to evaluate these categories for other survey years. Consistent with previous research,^8,19^ a top-code at the 98th percentile for debt was applied in each survey year to limit the influence of extreme values. All debt amounts were adjusted for inflation and reported in 2004 US$ to ensure comparability over time.

Unsecured debt trajectories were identified using a group-based trajectory model, which clustered respondents with similar longitudinal debt patterns. Group-based trajectory models use maximum likelihood estimation to identify shared trajectories among similar individuals and assign each respondent to a group based on the probability of correct placement.^21^ To ensure reliable assignment, a minimum value of 0·70 was required for the average posterior probability of assignment (APPA) for each group. The number of groups and the shape of the trajectories (polynomial order) were determined using Bayesian Information Criterion statistics to achieve the best data fit.^22^

The trajectory analysis identified a three-trajectory cubic model as the best fit, based on lower Bayesian Information Criterion statistics and higher APPA values compared with alternative models. The three identified trajectories were constant low debt, constant medium debt, and increasing debt. The average APPA for this model was 0·91, exceeding the 0·70 threshold recommended by Nagin,^21^ reinforcing the reliability of group assignment. A cubic polynomial was applied for all groups because the linear, quadratic, and cubic terms were significant in each case, capturing the complexity of debt trajectories. Additionally, a no debt group was manually included, defined as participants who consistently reported zero unsecured debt across the exposure period. Unsecured debt trajectories from 1985 to 2004 in 2004 US$ are included in the appendix (p 2).

### Covariates

We controlled for baseline variables collected in 1985 that are potential confounders for the association between unsecured debt and mortality, which were: baseline age (in years), sex or gender (men *vs* women), race or ethnicity (Black *vs* Hispanic *vs* other), own education (high school or higher *vs* lower than high school), parental education (high school or higher *vs* lower than high school), marital status (married *vs* other), employment status (unemployed *vs* employed or in school), and physical disability (yes *vs* no). Participant race or ethnicity was reported using categorisations used in NLSY79 documentation.^23^ Respondents’ net family income (reported in quartiles) and net family wealth (reported in quartiles) in 1985 were also controlled for. Total net family wealth was calculated by summing all the respondent’s family net assets and subtracting their total debts. Additionally, we controlled for health behaviours and other variables that are important risk factors for mortality and that were measured closest to baseline (1985), which included earlier life cognitive function, as assessed by Armed Forces Qualification Test (only available in 1981) with higher score indicating better cognition, BMI in 1985, smoking history in 1984 (ever smoked *vs* never smoked), and alcohol consumption (yes *vs* no) in the 30 days before the survey in 1985.

### Outcomes

The outcome of interest was premature mortality between 2004 and 2018. A participant was classified as deceased if their reason for not being interviewed in a given survey wave was listed as deceased, which meant the respondent was reported as deceased when interviewers attempted to make contact at that wave. These deaths were confirmed by NLSY79 staff by matching participant records to the National Center for Health Statistics National Death Index. Since NLSY79 did not record the exact date of death, the survey wave in which death was recorded was used as the individual’s year of death, and estimated month of death was estimated as the month of the survey in which death was recorded, and set the day of death as the first day of that month. Since individuals in this cohort would be aged 41–62 years during the follow-up period when the outcome of mortality was assessed, which is substantially lower than life expectancy from birth in the USA during this time,^1^ all deaths during the follow-up period were considered to be premature.

### Statistical analysis

We first described the distribution of sample characteristics overall and across the four unsecured debt trajectories. ANOVA was used for continuous variables and χ^2^ tests for categorical variables to test for differences in variables across strata of debt trajectory. For each trajectory group, the crude death rate per 10 000 person-years and incidence rate ratios (IRRs) were calculated, using the constant low debt group as the reference. To quantify excess risk attributable to debt trajectory, the crude difference in cumulative incidence between each debt trajectory group and the reference group (constant low debt) was also calculated. A Poisson approximation was used to estimate 95% CIs for incidence.

Cox proportional hazards regression models were fit to evaluate the association between unsecured debt trajectories and premature mortality, adjusted for covariates, computing hazard ratios (HRs) for each trajectory using the reference group of low unsecured debt. The Exact method was used for handling tie survival events, and proportional hazards assumption was checked using Schoenfeld residuals. Four Cox models were fit sequentially. Model 1 was unadjusted, model 2 was adjusted for age, race or ethnicity, sex or gender, own education and parents’ education, employment status, physical disability, and Armed Forces Qualification Test score. Model 3 was additionally adjusted for BMI, smoking history, and alcohol consumption, and model 4 was additionally adjusted for net family income and net family wealth. Survival curves were also generated, adjusted for all covariates (from model 4) to compare survival probability over time by unsecured debt trajectory. Person time was computed as the time between 2004 and death date (for decedents), study drop out at last year of interview (for individuals lost to follow-up between 2004 and 2018), or end of follow-up (for individuals who remained under observation and alive at the end of outcome follow-up).

As sensitivity analyses, we ran all regression models adjusting for the mean of covariates that varied over the exposure time, including average income, wealth, BMI, duration of marriage, duration of smoking, and duration of drinking, along with other fixed covariates. In an additional sensitivity analysis, all regression models were also run using three trajectories of unsecured debt exposure, combining the constant low unsecured debt and constant medium unsecured debt groups due to their similar debt patterns and minimal difference in mortality risk in analyses. Total unstructured debt (ie, not only our analytic sample) in NLS79 was compared with unstructured debt among all American adults, and all American adults aged 35–44 years, using national data obtained from the US Census^24^ for years 2000 and 2004 (ie, the years that overlapped with the study exposure period). Additionally, trajectory models and unadjusted analysis were repeated on a sample of participants from NLSY79 with missing covariates, to assess whether missingness had an effect on the analysis results. The main adjusted analysis was also repeated to include covariates for hypertension and diabetes measured at age 40 years (measured between 1998 and 2006). In our analysis, NLSY79 survey weights were not used, since the NLSY79 advises caution when using these weights for regression analysis,^25^ and previous research has questioned the utility of using survey weights in regression analysis with subsets of data.^26^ All analyses were conducted in R (version 4.4.1) with dplyr, tidyverse, survival, survminer, and timereg packages.

### Role of the funding source

The funder of the study had no role in study design, data collection, data analysis, data interpretation, or writing of the report.

## Results

Our analytic sample consisted of 6954 participants. Between years 1985 and 2004, 5670 (81·5%) of 6954 individuals had constant low unsecured debt, 712 (10·2%) had constant medium unsecured debt, 148 (2·1%) had increasing unsecured debt, and 424 (6·1%) had no unsecured debt (table 1). The mean age of participants was 24·4 years (SD 2·2) in 1985, and 3651 (52·5%) of 6954 participants of the sample were women. Individuals in the no debt group were more likely to be Black, have less than a high school education, be unemployed, and havelower mean income and wealth (table 1).

**Figure 1:**
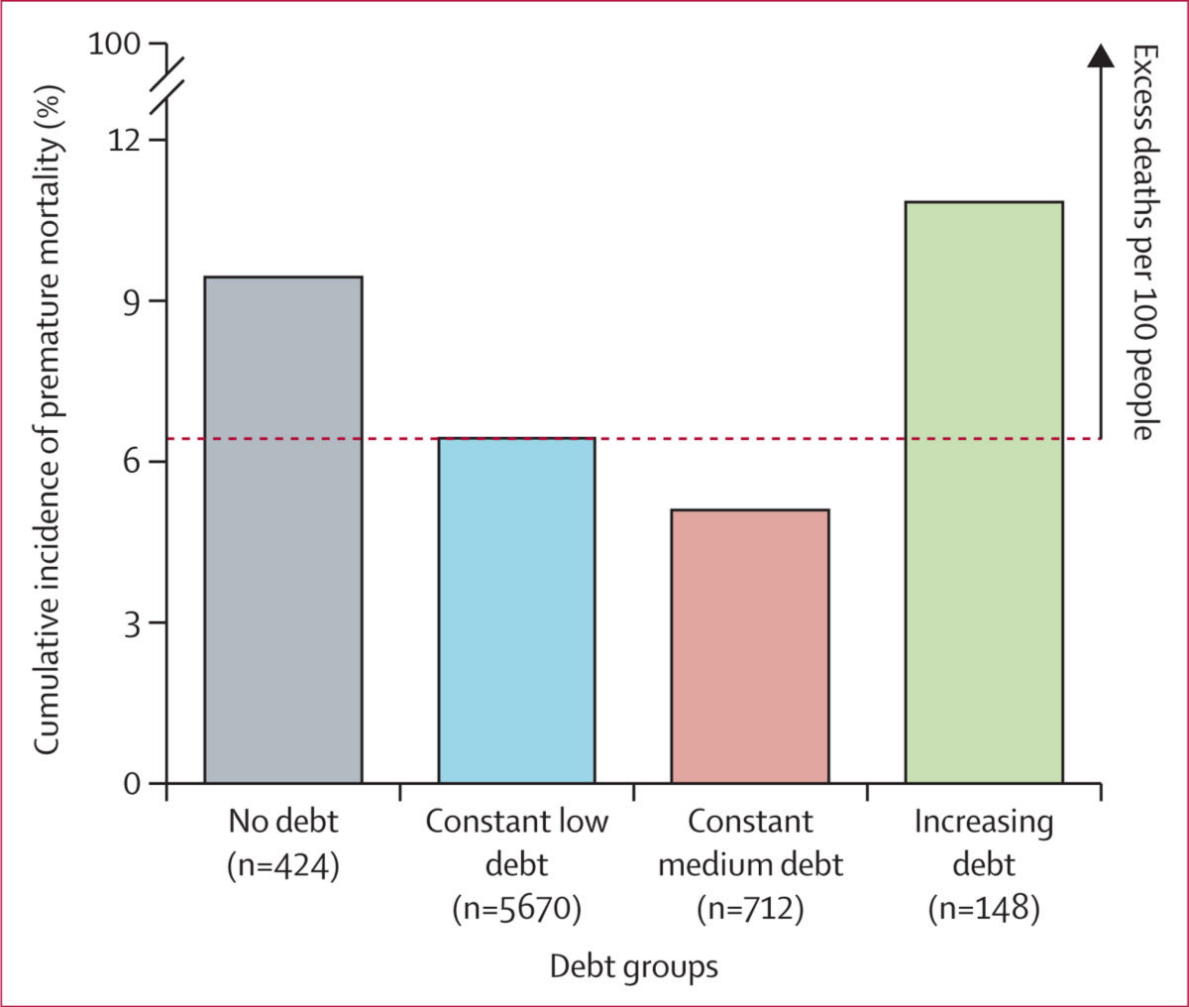
Overall cumulative incidence of premature mortality (2004–18) across trajectories of unsecured debt (1985–2004) in the National Longitudinal Survey of Youth 1979 (n=6954) The overall cumulative incidence rate for each trajectory group was calculated by dividing the total number of deaths by the total number of participants in each trajectory group and then multiplying by 100.

In our crude analysis (table 2), individuals in the increasing debt group had a 79% higher unadjusted risk of mortality than individuals in the constant low debt group (IRR 1·79 [95% CI 1·13–2·62]). Additionally, compared with individuals in the constant low debt group, individuals with no debt had a 52% higher risk of premature mortality (1·52 [1·21–1·87]). The number of excess deaths attributable to debt was higher among the increasing debt and no debt groups than the constant low debt and constant medium debt groups over time (figure 1).

**Figure 2:**
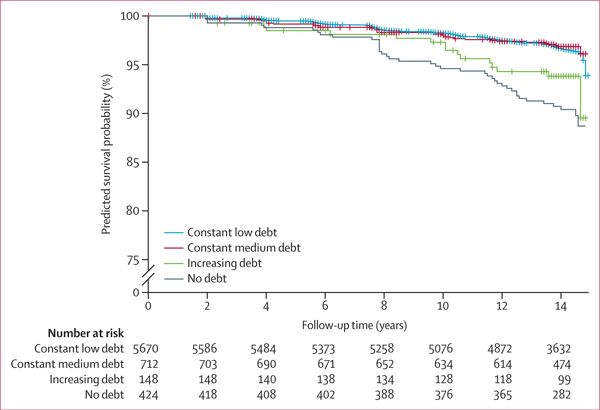
Predicted survival probability during follow-up (2004–18) by unsecured debt trajectory (1985–2004) in the National Longitudinal Survey of Youth 1979 (n=6954) Adjusted for age in 1985, sex, race, marital status in 1985, respondents’ highest education completed in 1985, respondents’ parent’s highest education completed in 1985, employment status in 1985, Armed Forces Qualification Test score in 1981, BMI in 1985, smoking in 1985, alcohol consumption in 1985, net family income in 1985, and net family wealth in 1985.

In our minimally adjusted Cox proportional hazards model (table 3), individuals in the increasing debt group had a 94% higher risk of premature mortality than individuals in the constant low debt group (HR 1·94 [95% CI 1·17–3·21]). This association only slightly decreased and remained significant in the fully adjusted model (model 4; 1·89 [1·14–3·12]). In unadjusted models, individuals in the no debt group had a 48% higher risk of premature mortality than individuals in the constant low debt group (1·48 [1·07–2·05]). This association was attenuated and no longer significant in the fully adjusted model (1·28 [0·91–1·81]). The point estimate suggested an increased risk of premature mortality associated with no debt when compared with constant low debt in the fully adjusted model. Fully adjusted survival analysis indicated that individuals in the no debt and increasing debt groups had worse survival probabilities than did individuals in the constant low and constant medium debt groups (figure 2).

In sensitivity analysis (appendix p 3) adjusted for the mean of covariates over the exposure period, results were similar in terms of direction and magnitude to the main analysis. The sensitivity analysis examining three categories of debt (ie, combining the low and medium unsecured debt trajectories) was also similar in magnitude and direction when comapared with the main analysis (appendix p 4), as was the sensitivity analysis including covariates for diabetes and hypertension measured at age 40 years (appendix p 5). When the unadjusted analysis was repeated to include participants with missing covariate data, the results were also similar in magnitude and direction (appendix p 6). When the amount of unstructured debt held by individuals in the sample was compared with national data in 2004, individuals included in this sample had less unsecured debt (appendix p 7).

## Discussion

In fully adjusted models, individuals with increasing unsecured debt in early adulthood had an 89% higher risk of premature mortality risk in midlife when compared with individuals with constant low unsecured debt. This finding was robust to adjustments for sociodemographic characteristics and health behaviours across several models.

Our results build on previous studies,^16–18^ which found that debt was associated with mortality. Our results provide strong evidence for this association in a nationally representative longitudinal cohort study, and using a long trajectory of cumulative debt in early adulthood to midlife as the exposure. Our findings are consistent with other longitudinal cohort studies and a preprint^27^ on the association between measures of debt and various types of mortality, including suicide mortality,^12^ and alcohol-related mortality,^28^ which are both drivers of premature death in the USA.^3,29^ Our findings are also consistent with other studies showing that early adulthood trajectories of related social determinants of health (occupation and income) are associated with premature mortality in midlife.^30,31^

Individuals with no unsecured debt had an increased risk and excess premature mortality in the unadjusted analysis, and the point estimate indicated increased risk in adjusted analysis, although this difference was not statistically significant. In our sample, it might be that people with no debt were unable to take on business loans or credit cards, or did not have medical debt because they were entirely excluded from accessing health care. Individuals with no debt in our study were more likely to be Black Americans, and to have lower education amd less income and wealth, which are groups that have historically had limited access to financial resources. Our findings are also consistent with previous research showing that individuals with a low income increasingly lack access to credit cards, and that there are increasing restrictions that might prevent Americans with a low income from accessing credit cards.^32^ In this sense, trajectories of debt might reflect the different social systems people had access to in early adulthood, highlighting the importance of carefully considering what debt, or the absence of debt, means as a social determinant of health. We recommend future research further examines more complex constructs of debt, considering that different types of debt (rather than the presence or absence of debt) might be key in understanding how debt is associated with health outcomes.

Several mechanisms could mediate the association between unsecured debt in early life and premature midlife mortality. Debt might be associated with premature mortality through pathways related to mental health,^9,12,14,15^ physical health,^8–12^ and health-care access.^33^ Some of these pathways might be specific to the type of unsecured debt—eg, medical debt being a barrier to health-care access.^33^ Overall, considering the strong association between debt and mental health outcomes such as depression^15^ and suicide attempts,^12^ and that mental health-related mortality outcomes including suicide, drug overdose, and alcohol use are strong drivers of premature mortality in the USA,^29^ it is likely that mental health is a key pathway in this association. We believe this association could be bidirectional: debt could possibly lead to mental illness, while mental illness could also lead to financial precarity and thus increased debt. Debt is also associated with financial insecurity in other domains, including food and housing insecurity,^34^ which are in turn determinants of premature mortality.^35^ In addition to the direct material consequences of debt, qualitative research highlights the ways in which high amounts of debt are associated with feelings of guilt, shame, anxiety, and stress, which might contribute to the association between debt and health.^36,37^

There are several existing interventions that might prevent the possible negative health consequences of unsecured debt on health, including behavioural interventions such as financial counselling,^38,39^ and policies that might prevent people from accruing unsecured debt.^40,41^ Financial counselling interventions to improve debt management have been shown to improve financial wellbeing^39^ and health outcomes.^38^ At the policy level, there is evidence that policies restricting the interest rates that might be charged by payday lenders might reduce mortality related to suicide and drug overdose, as shown in a prepint study,^41^ and premature mortality overall.^40^ Policies that improve financial wellbeing among individuals of low socioeconomic status have also been shown to reduce certain types of unsecured debt. For example, policies expanding access to health insurance are shown to reduce medical debt,^42,43^ while a preprint^44^ showed that policies that raise minimum wages reduce the use of high interest borrowing such as payday loans.^44^ Considering the results of our longitudinal analysis, we advocate for the use of these policies and interventions.

Our study has limitations. Our increasing unsecured debt group was relatively small, and thus might not be representative of more common experiences of debt in the USA. When comparing debt in our sample to national data on individuals aged 35–44 years in the USA in 2004, individuals in our sample had less debt. Another limitation is that due to the small size of this exposure group, we were unable to stratify our results by sex and race or ethnicity constructs, which is useful for understanding health inequalities. Despite the small sample size of the increasing debt group, our analysis was statistically powered to detect the association identified between unsecured debt and premature mortality. However, it is possible that due to the size of this group outlier datapoints could have led to biased results. Much of the debt accrued by individuals in the increasing unsecured debt group was accumulated in the fourth decade of life, which is a time in life when family and medical expenses might become more over-whelming. We recommend that future analyses extend follow-up further into midlife, and also examine cohorts who were in early adulthood in times of greater financial uncertainty, such as the Great Recession. Survey data are subject to recall bias, and therefore it is possible that survey participants did not understand the question or chose not to disclose their true financial status. The questions on unsecured debt could also be misconstrued as referring to some types of secured debt. Consistent with previous research,^37^ we recommend that future research include more carefully crafted and nuanced debt questions, with special attention to different types of debt, being unable to pay debt, and being unable to access wealth building debt systems. Additionally, we did not have access to the linked data between the NLSY79 and the National Death Index and therefore we were not able to ascertain the association between debt and cause-specific mortality. It is unlikely that we would have been powered to detect associations with specific causes of mortality. It is likely that the causes of premature mortality in our sample are similar to the leading causes of premature mortality nationwide during this time (suicide, alcohol use, and drug overdose).^3,29^ We did not have access to actual date of death for our analysis, and instead used survey year of death to approximate date of death, which could have resulted in non-differential misclassification of outcome, thereby underestimating the true association between debt and premature mortality. The findings of our sensitivity analysis in which additional adjustments for diabetes and hypertension status were made were consistent with our main analysis. It is important to note that our exposure period began at a time in early life when the sample might have had few comorbidities, and the NLSY79 did not begin collecting data on comorbidities until participants were aged 40 years. Adjusting for comorbidities at this time might reflect that these conditions are mediators in the causal pathway from debt to mortality, rather than confounding variables that would be common causes of both debt and mortality. For this reason, we consider our models adjusting for the early life health risk behaviours (smoking, BMI, and alcohol use) to be our main analysis. Another approach to evaluating the association between debt and health is longitudinal panel modelling, which could investigate research questions on both short-term and long-term associations between debt and mortality. We recommend that future research consider these types of models. Finally, although previous research has emphasised the importance of credit card debt,^11^ due to the limitation of NLSY79 survey design, we were unable to separate consumer debt, debt owed to others, or other categories from the overall unsecured debt total.

Our study has several notable strengths. This study is one of few cohort studies to examine trajectory of debt as the exposure,^8,13,19^ which provides stronger evidence than other studies. To our knowledge, our study is the first to examine the association between trajectory of debt and premature mortality. The NLSY79 sample provided more than 30 years of total follow-up data, with more than 20 years of exposure follow-up data, and 12 years of outcome follow-up data, providing strong evidence of this association. The NLSY79 collected rich data on debt, which allowed us to focus on unsecured debt, while previous studies have used less precise measurements of debt. Our findings were robust across models and in two sensitivity analyses, strengthening our confidence in these results.

The findings of our study demonstrate that cumulative increasing unsecured debt in early adulthood is associated with an elevated risk of premature mortality in midlife. Additionally, having no debt might reflect social and financial exclusion rather than financial stability, reinforcing the need to consider both the absence of debt and increasing debt or debt accumulation as relevant social determinants of health. Unsecured debt in the USA is increasing.^6,45^ Emerging financial technology offers new methods for low credit score consumers to access unsecured loans, which has driven an increase in unsecured personal loan balances in the USA since 2020.^45^ Considering that Americans at present hold a much larger sum of some types of unsecured debt than they did during our study period,^6^ our findings and recommendations might be even more relevant. Several interventions and policies for preventing individuals from accruing or continuing to accrue unsecured debt currently exist. We recommend future research focus on the evaluation and development of these interventions and policies, and that public health practitioners advocate for the immediate use of existing policies and interventions for preventing debt-related premature mortality in the USA.

## Supplementary Material

1

## Figures and Tables

**Table 1:** Baseline characteristics of NLSY79 study participants (1985–2018; n=6954)

	Overall (n=6954)	Constant low debt (n=5670)	Constant medium debt (n=712)	Increasing debt (n=148)	No debt (n=424)	p value
Age, years	24·4 (2·2)	24·3 (2·2)	24·5 (2·2)	24·4 (2·2)	24·3 (2·2)	0·20
Race or ethnicity	··	··	··	··	··	0·20
Black	2046 (29·4%)	1689 (29·8%)	120 (16·9%)	27 (18·2%)	210 (49·5%)	··
Hispanic	1288 (18·5%)	1103 (19·5%)	110 (15·4%)	27 (18·2%)	48 (11·3%)	··
Other	3620 (52·1%)	2878 (50·8%)	482 (67·7%)	94 (63·5%)	166 (39·2%)	··
Sex	··	··	··	··	··	0·37
Female	3651 (52·5%)	2997 (52·9%)	373 (52·4%)	76 (51·4%)	205 (48·3%)	··
Male	3303 (47·5%)	2673 (47·1%)	339 (47·6%)	72 (48·6%)	219 (51·7%)	··
Education	··	··	··	··	··	<0·0001
Less than high school	1174 (16·9%)	959 (16·9%)	64 (9·0%)	24 (16·2%)	127 (30·0%)	··
High school or higher	5780 (83·1%)	4711 (83·1%)	648 (91·0%)	124 (83·7%)	297 (70·0%)	··
Parents’ education	··	··	··	··	··	<0·0001
Less than high school	2362 (34·0%)	1986 (35·0%)	152 (21·3%)	42 (28·6%)	182 (42·9%)	··
High school or higher	4592 (66·0%)	3684 (65·0%)	560 (78·7%)	106 (71·4%)	242 (57·1%)	··
Marital status	··	··	··	··	··	<0·0001
Married	2462 (35·4%)	2037 (35·9%)	282 (39·6%)	61 (41·4%)	82 (19·3%)	··
Other	4492 (64·6%)	3633 (64·1%)	430 (60·4%)	87 (58·5%)	342 (80·7%)	··
Employment status	··	··	··	··	··	<0·0001
Employed or in school	5298 (77·6%)	4325 (76·3%)	611 (85·8%)	119 (80·3%)	243 (57·3%)	··
Unemployed or not in school	1656 (23·8%)	1345 (23·7%)	101 (14·2%)	29 (19·7%)	181 (42·7%)	··
Household income, US$	19 400 (19 000)	19 200 (18 600)	22 900 (21 900)	20 900 (18 500)	15 200 (17 200)	<0·0001
Household wealth, US$	10 200 (42 600)	9970 (42 000)	13 100 (54 100)	12 900 (45 700)	7440 (23 400)	0·12
Smoking status	··	··	··	··	··	0·0009
Ever smoked	5573 (80·1%)	4557 (80·4%)	580 (81·5%)	126 (85·0%)	310 (73·1%)	··
Never smoked	1381 (19·9%)	1113 (19·6%)	132 (18·5%)	22 (15·0%)	114 (26·9%)	··
Alcohol consumption in the previous month	··	··	··	··	··	<0·0001
Yes	4663 (67·1%)	3800 (67·0%)	521 (73·2%)	105 (70·8%)	237 (55·9%)	··
No	2291 (32·9%)	1870 (33·0)	191 (26·8%)	43 (29·3%)	187 (44·1%)	··
BMI, kg/m^2^	23·9 (4·2)	23·9 (4·2)	23·7 (4·0)	23·8 (4·4)	23·8 (4·1)	0·45
AFQT percentile score	41·1 (29·6)	40·3 (28·9)	53·6 (30·2)	47·9 (30·1)	28·2 (29·8)	<0·0001
Unsecured debt (1985), US$	1890 (6880)	1330 (3050)	6960 (17 900)	4350 (13 500)	0	<0·0001
Unsecured debt (2004), US$	4880 (14 100)	3070 (5390)	12 300 (22 100)	52 300 (56 300)	0	<0·0001

Data are mean (SD) or n (%). All characteristics were captured at baseline for this analysis (1985), with the exception of smoking status, which was collected in 1984, and the AFQT, which was collected in 1981, as part of NLSY79. Household income and wealth are reported in 2004 US$. p values were calculated using ANOVA for continuous variables and χ^2^ tests for categorical variables. AFQT=Armed Forces Qualification Test. NLSY79=National Longitudinal Survey of Youth 1979.

**Table 2:** Incidence of premature mortality and excess deaths overall across trajectories of unsecured debt in the National Longitudinal Survey of Youth 1979 (1985–2018; n=6954)

	Participants, n	Deaths, n	Person-years	Deaths per 10 000 person-years (95% CI)	Excess deaths per 10 000 person-years (95% CI)	Unadjusted incidence rate ratio (95% CI)
Overall	6954	455	603	48·1 (43·8–53·0)	··	··
Debt trajectory Constant low debt	5670	363	355	46·9 (42·2–52·0)	(ref)	(ref)
Constant medium debt	712	36	9694	37·1 (26·0–51·4)	–9·8 (–16·2 to –0·6)	0·79 (0·62–0·99)
Increasing debt	148	16	1908	83·9 (47·9–136·2)	37·0 (5·7 to 84·2)	1·79 (1·13–2·62)
No debt	424	40	5590	71·5 (51·0–97·4)	24·6 (8·9 to 45·4)	1·52 (1·21–1·87)

**Table 3:** Associations between trajectories of unsecured debt (1985–2004) and premature mortality (2004–18) in the National Longitudinal Survey of Youth 1979 (n=6954)

	Model 1	Model 2	Model 3	Model 4
Constant low debt	1 (ref)	1 (ref)	1 (ref)	1 (ref)
Constant medium debt	0·79 (0·56–1·12)	0·92 (0·66–1·31)	0·93 (0·66–1·31)	0·91 (0·64–1·29)
Increasing debt	1·74* (1·06–2·88)	1·94* (1·17–3·21)	1·90* (1·15–3·15)	1·89* (1·14–3·12)
No debt	1·48* (1·07–2·05)	1·18 (0·84–1·64)	1·25 (0·89–1·75)	1·28 (0·91–1·81)

Data are hazard ratio (95 % CI). Model 1 was unadjusted. Model 2 was adjusted for age in 1985, sex, race, marital status in 1985, respondents’ highest education completed in 1985, respondents’ parents’ highest education completed in 1985, employment status in 1985, and Armed Forces Qualification Test score in 1981. Model 3 was additionally adjusted for BMI in 1985, smoking in 1985, and alcohol consumption in 1985. Model 4 was additionally adjusted for net family income in 1985, and net family wealth in 1985.

* p<0·05.

## Data Availability

The data used in this study are publicly available from the US Bureau of Labor Statistics website (https://www.bls.gov/nls/nlsy79.htm), where de-identified data with a data dictionary are available. Study authors of this manuscript will share our statistical code for this analysis with any interested party on request to az2567@cumc.columbia.edu.

## Research in context

### Evidence before this study

We searched PubMed from database inception to Feb 15, 2025, for studies using the comprehensive terms “unsecured debt” AND “health”. No language restrictions were applied. Our search yielded 17 articles. Of these articles, many were economic and epidemiological studies, including a meta-analysis of epidemiological studies, which found strong associations between unsecured debt and many health outcomes, particularly mental health outcomes. Of these studies, only a small number used a life course approach to assess longitudinal exposures and cumulative debt trajectories, and no studies included premature mortality as an outcome.

### Added value of this study

To our knowledge, this is the first study to demonstrate statistically significant associations between an early adulthood trajectory of increasing unsecured debt and premature mortality in midlife (age 41–62 years). Although no significant differences were identified in adjusted models, individuals with no debt had a higher risk of premature mortality than individuals with low debt in unadjusted analysis. Having no debt might reflect social and financial exclusion rather than financial stability; therefore, these findings suggest that both the absence of debt and increasing debt or debt accumulation might be important social determinants of health.

### Implications of all the available evidence

Together, these studies show that increasing unstructured debt in early adulthood is an important determinant of health outcomes, including premature mortality. Several interventions and policies for preventing the accrual or continued accrual of unsecured debt currently exist, such as financial counselling and policies to prevent predatory lending. Future research should focus on the evaluation and development of these interventions and policies, and public health practitioners should advocate for the immediate use of existing policies and interventions for preventing debt-related premature mortality.
