# Supplementary material for: Unsecured debt in early adulthood and premature mortality in adults in the USA: a longitudinal analysis of prospective national cohort data
Source: Lancet Public Health. Author manuscript; Available in PMC 2025 Dec 12. (PMC12700172; doi:10.1016/S2468-2667(25)00226-9)
Supplement: 1 [file NIHMS2119843-supplement-1.pdf]

THE LANCET
Public Health

Supplementary appendix

This appendix formed part of the original submission and has been peer reviewed. We post it as supplied by the authors.

Supplement to: Swift SL, Chen Z, Colvin C, Kezios K, Calonico S, Zeki Al Hazzouri A. Unsecured debt in early adulthood and premature mortality in adults in the USA: a longitudinal analysis of prospective national cohort data. *Lancet Public Health* 2025; **10:** e979–87.

SUPPLEMENTARY MATERIAL

Supplemental Figure 1. Study Sample Flowchart, NLSY 1979

Supplemental Figure 2. Average amount of unsecured debt (in 2004 Dollars) by debt trajectory and survey year, NLSY79 study, N=6,954

Supplemental Table 1. Hazard ratios from cox proportional hazards regression models for the association between trajectories of unsecured debt (1985 – 2004) and premature mortality (2004 – 2018), adjusting for the average of time-varying covariates over exposure period, NLSY79 Study, N=6,954

Supplemental Table 2. Hazard ratios from cox proportional hazards regression models for the association between trajectories of unsecured debt (1985 – 2004) and premature mortality (2004 – 2018), using a 3-trajectory model combining constant low and medium debt trajectories, NLSY79 Study, N=6,954

Supplemental Table 3. Hazard ratios from cox proportional hazards regression models for the association between trajectories of unsecured debt (1985 – 2004) and premature mortality (2004 – 2018), NLSY79 Study, N=6,954

Supplemental Table 4. Premature Mortality Incidence Rate Ratios and Excess Deaths Overall and Across Trajectories of Unsecured Debt, including participants with missing covariate data NLSY79 Study, 1985-2018, N=7,452.

Supplemental Table 5. Comparison of Average Unsecured Debt in the NLSY79 Study Sample with the U.S. National Averages (in 2004 dollars)

Supplemental Figure 1. Study Sample Flowchart, NLSY 1979

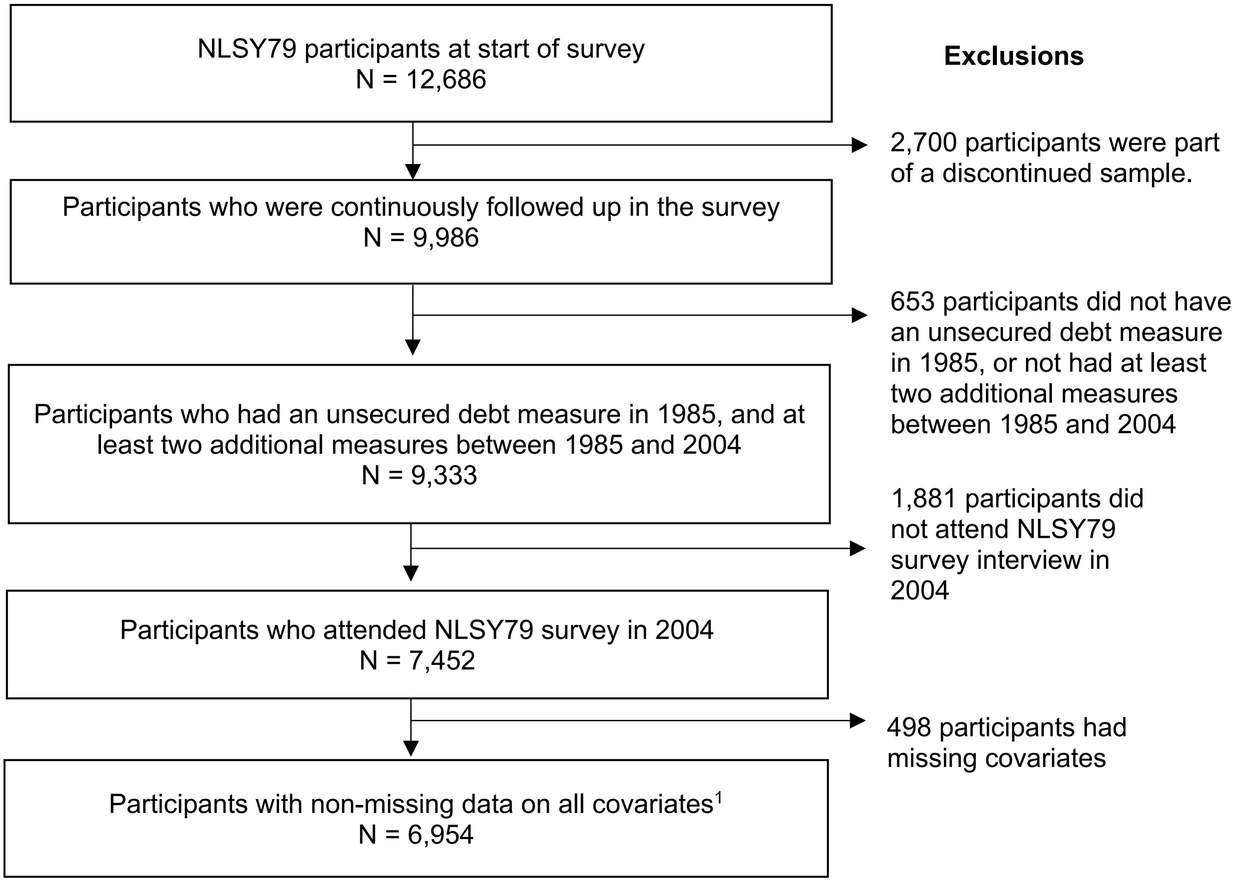

NLSY79: the National Longitudinal Survey of Youth 1979
[1]Covariates defining the complete case sample included: age in 1985, race/ethnicity, gender, marital status in 1985, respondents' highest education, respondents' parents' highest education, employment status in 1985, baseline cognition, body mass index (BMI) in 1985, smoking status in 1985, alcohol consumption in 1985, net family income in 1985, and net family wealth in 1985

Supplemental Figure 2. Average amount of unsecured debt (in 2004 Dollars) by debt trajectory and survey year, NLSY79 study, N=6,954

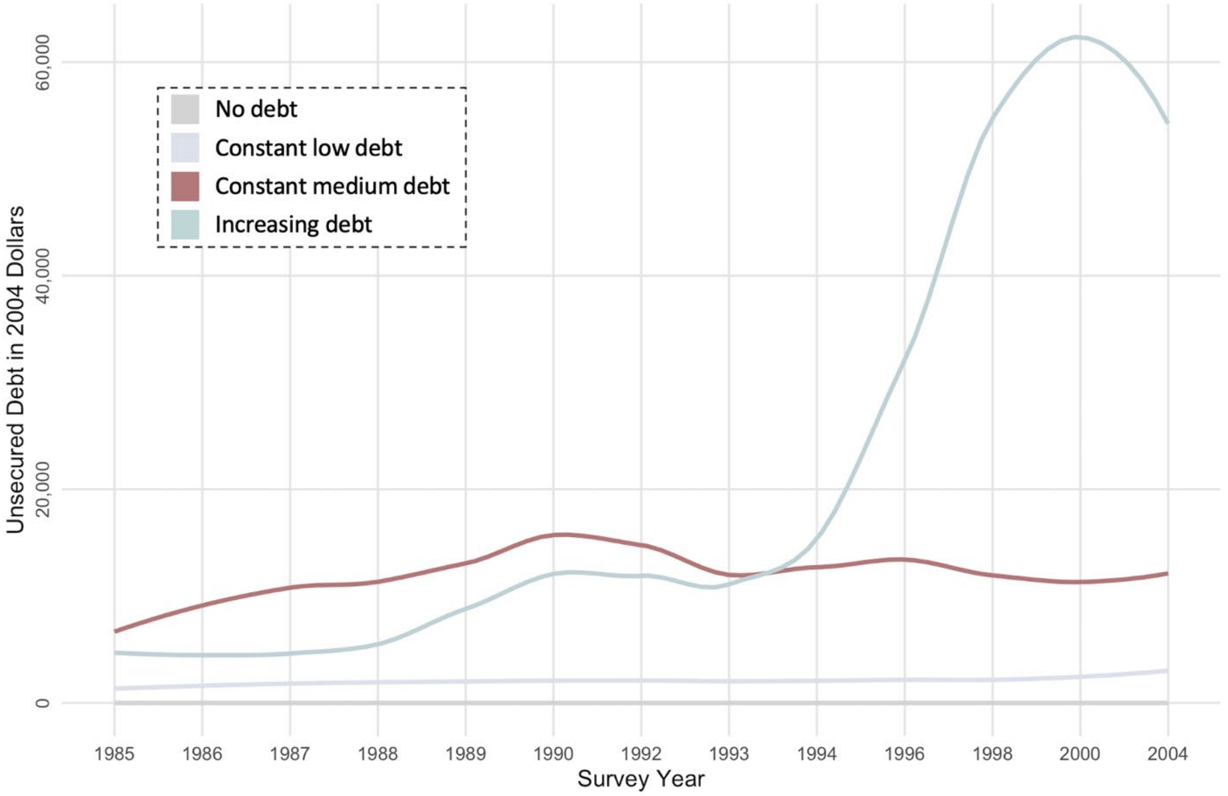

Supplemental Table 1. Hazard ratios from cox proportional hazards regression models for the association between trajectories of unsecured debt (1985 – 2004) and premature mortality (2004 – 2018), adjusting for the average of time-varying covariates over exposure period, NLSY79 Study, N=6,954

	**Model 1**	**Model 2**	**Model 3**	**Model 4**
	HR (95% CI)	HR (95% CI)	HR (95% CI)	HR (95% CI)
**Constant low debt**	Referent	Referent	Referent	Referent
**Constant medium debt**	0.79<br>(0.56, 1.12)	0.95<br>(0.67, 1.35)	0.95<br>(0.67, 1.34)	0.96<br>(0.67, 1.35)
**Increasing debt**	1.74*<br>(1.06, 2.88)	1.96*<br>(1.19, 3.25)	1.94*<br>(1.17, 3.22)	1.92*<br>(1.15, 3.18)
**No debt**	1.48*<br>(1.07, 2.05)	1.02<br>(0.73, 1.42)	1.13<br>(0.80, 1.58)	1.14<br>(0.81, 1.61)

Model 1 is unadjusted; Model 2 is adjusted for age in 1985, gender, race, respondents' highest education completed in 1985, respondents' parent's highest education completed in 1985, AFQT score in 1981, percentage of marriage duration from 1985 to 2004, and percentage of employment duration from 1985 to 2004. Model 3 is additionally adjusted for average BMI from 1985 to 2004, percentage of smoking duration from 1985 to 2004, and percentage of alcohol consumption duration from 1985 to 2004. Model 4 is additionally adjusted for average net family income from 1985 to 2004, and average net family wealth from 1985 to 2004.
*Statistically significant, $p < 0.05$

Supplemental Table 2. Hazard ratios from cox proportional hazards regression models for the association between trajectories of unsecured debt (1985 – 2004) and premature mortality (2004 – 2018), using a 3-trajectory model combining constant low and medium debt trajectories, NLSY79 Study, N=6,954

	**Model 1**	**Model 2**	**Model 3**	**Model 4**
	HR (95% CI)	HR (95% CI)	HR (95% CI)	HR (95% CI)
**Three debt trajectories**				
**Constant low/medium**	Referent	Referent	Referent	Referent
**Increasing debt**	1.78* (1.08, 2.94)	1.96* (1.18, 3.23)	1.92* (1.16, 3.17)	1.91* (1.15, 3.15)
**No debt**	1.51* (1.09, 2.09)	1.18 (0.85, 1.65)	1.26 (0.90, 1.76)	1.29 (0.92, 1.82)

Model 1 is unadjusted. Model 2 is adjusted for age in 1985, gender, race, marital status in 1985, respondents' highest education completed in 1985, respondents' parent's highest education completed in 1985, employment status in 1985, and AFQT score in 1981. Model 3 is additionally adjusted for BMI in 1985, smoking in 1985, alcohol consumption in 1985. Model 4 is additionally adjusted for net family income in 1985, and net family wealth in 1985.
*Statistically significant, $p < 0.05$

Supplemental Table 3. Hazard ratios from cox proportional hazards regression models for the association between trajectories of unsecured debt (1985 – 2004) and premature mortality (2004 – 2018), NLSY79 Study, N=6,954

	**Model 1**	**Model 2**	**Model 3**	**Model 4**
	**HR(95% CI)**	**HR (95% CI)**	**HR (95% CI)**	**HR (95% CI)**
**Debt trajectory**				
**Constant low debt**	Referent	Referent	Referent	Referent
**Constant medium debt**	0.79 (0.56, 1.12)	0.92 (0.66, 1.31)	0.93 (0.66, 1.31)	0.89 (0.63, 1.27)
**Increasing debt**	1.74* (1.06, 2.88)	1.94* (1.17, 3.21)	1.90* (1.15, 3.15)	1.74* (1.05, 2.89)
**No debt**	1.48* (1.07, 2.05)	1.18 (0.84, 1.64)	1.25 (0.89, 1.75)	1.30 (0.93, 1.82)

Model 1 is unadjusted.
Model 2 is adjusted for age in 1985, gender, race, marital status in 1985, respondents' highest education completed in 1985, respondents' parent's highest education completed in 1985, employment status in 1985, and AFQT score in 1981.
Model 3 is additionally adjusted for BMI in 1985, smoking in 1985, alcohol consumption in 1985.
Model 4 is additionally adjusted for net family income in 1985, net family wealth in 1985, diabetes and hypertension measured at age 40, between 1998 and 2006
*Statistically significant, $p < 0.05$

Supplemental Table 4. Premature Mortality Incidence Rate Ratios and Excess Deaths Overall and Across Trajectories of Unsecured Debt, including participants with missing covariate data NLSY79 Study, 1985-2018, N=7,452.

	N	Deaths	Person - Years (PY)	Death per 10 000 PY (95% CI)	Excess deaths per 10 000 PY (95% CI)	Unadjusted Incidence Rate Ratio (95% CI)
**Overall**	7,452	499	98,229	50.7 (46.4 to 55.5)	-	-
**Debt trajectory**						
Constant low debt	6,102	400	80,494	49.6 (44.9 to 54.8)	Referent	Referent
Constant medium debt	753	41	9,896	41.4 (29.7, 56.2)	-8.2 (-21.7, -5.4)	0.84 (0.60, 1.15)
Increasing debt	173	18	2,249	80.0 (47.4, 126.5)	30.4 (-6.9, 67.7)	1.62 (1.00, 2.58)
No debt	424	40	5,590	71.5 (51.1, 97.4)	21.9 (-8.0, 44.6)	1.44 (1.04, 1.99)

Supplemental Table 5. Comparison of Average Unsecured Debt in the NLSY79 Study Sample with the U.S. National Averages (in 2004 dollars)

Year	NLSY79 analytical sample	U.S. total	35 to 44 years old in U.S.
**2000**	4,455	10,678	11,550
**2004**	4,882	14,230	15,123

All amounts were adjusted to 2004 dollars for consistency. Data on average unsecured debt for U.S. individuals, both across all age groups and specifically for those aged 35 to 44 (the age range of our sample during the exposure period), was sourced from the U.S. Census Bureau's Survey of Income and Program Participation. The Census Bureau provides data on unsecured debt from 2000 to 2022, with the only overlap between their data and our exposure period occurring in the years 2000 and 2004.
